# Effectiveness and Cost Analysis of Implementation of Tele-ICU: A Multicenter Administrative Database Study Using Interrupted Time-Series Analysis Within a University-Affiliated Hospital Network

**DOI:** 10.3390/jcm15114316

**Published:** 2026-06-03

**Authors:** Kazuki Kikuyama, Hiroyuki Ohbe, Yumi Igarashi, Taro Watanabe, Maiko Mori, Toshikazu Abe, Toru Kotani

**Affiliations:** 1 Department of Intensive Care Medicine, Showa Medical University, Tokyo 142-8666, Japan; 47igayumi@gmail.com (Y.I.); watanabe-tr@med.showa-u.ac.jp (T.W.); sawamai.11.156@gmail.com (M.M.); abetoshi1@med.showa-u.ac.jp (T.A.); trkotani@med.showa-u.ac.jp (T.K.); 2 Department of Emergency and Critical Care Medicine, Tohoku University Hospital, Sendai 980-8574, Japan; hohbey@gmail.com

**Keywords:** critical care, telemedicine, medical cost, DPC system

## Abstract

**Background:** The telemedicine intensive care unit (tele-ICU) enables remote monitoring and support for intensive care unit (ICU) patients, connecting onsite care teams with remote critical care specialists through audio–video links and electronic data. While it has been shown to improve outcomes and reduce costs in the U.S., evidence on tele-ICU in Japan is limited. The present study aimed to investigate the effectiveness and cost of the implementation of tele-ICU in Japanese hospitals. **Methods:** This retrospective cohort study used administrative data from two Japanese hospitals where tele-ICU was implemented in April 2018. All adult patients admitted to the ICUs from April 2014 to March 2022 were included. Interrupted time-series analysis was conducted to compare trends in in-hospital mortality, complications, and hospitalization costs before and after tele-ICU implementation, with adjustments for baseline characteristics. **Results:** A total of 26,684 patients were included. Of these, 10,981 (41%) and 15,703 (59%) were before and after tele-ICU implementation, respectively. After tele-ICU implementation, there were significant downward level changes for in-hospital mortality (−2.62%; 95% confidence intervals −4.08 to −1.16%) and hospitalization costs (−221 thousand yen per patient; −412 to −29.7). A significant downward slope change was observed for complications (−1.82% per year; −2.54 to −1.09%). Subgroup analysis revealed that the mortality reduction was observed mainly in medium-severity, nonsurgery, and general ICU patients. **Conclusions:** Tele-ICU implementation was associated with lower in-hospital mortality, fewer complications, and lower hospitalization costs, particularly among medium-severity and nonsurgical patients. Further multicenter prospective studies are warranted.

## 1. Introduction

The telemedicine intensive care unit (tele-ICU) is a modern technology that involves intensive care professionals remotely supporting critically ill patients and onsite ICU staff through secure audio–video and electronic links [1]. This system allows intensivists or critical care specialists to monitor and treat multiple ICU patients simultaneously from other remote locations around the clock. The coronavirus disease 2019 pandemic accelerated the adoption of telemedicine, highlighting its potential and the need for its integration into the healthcare system [2].

The tele-ICU system was first reported in the United States in 1977, demonstrating its potential effectiveness and feasibility in improving the quality of critical care [3], which subsequently triggered its development and widespread adoption. Of particular interest is the effect on clinical indicators such as patient outcomes and economic outcomes. An observational study [4] of tele-ICU implementation at seven adult ICUs in the United States revealed that tele-ICU implementation was significantly associated with a reduction in mortality and length of stay in the ICU (ICU-LOS) and standardization of care, with adjustment for Acute Physiology and Chronic Health Evaluation score version IV (APACHE IV) and other variables. Furthermore, this study reported that the tele-ICU could be more effective at reducing mortality under low-intensity conditions, such as at nighttime rather than in the daytime. Another single-center observational study revealed that the implementation of tele-ICU increased the annual case volume by 20% and increased ICU revenue from approximately US$7.92 million to US$37.67 million [5].

These previous studies were conducted mainly in the United States, where the provisions for critical care, authority for interventions, staffing, and number of intensivists differed from those in Japan [6,7,8]. In addition, the demographic characteristics and distributions of inpatients receiving critical care in both countries vary widely [9]. Therefore, the effectiveness of the tele-ICU in these previous studies could not be applied directly to Japan. Our previous study evaluated the effectiveness of the tele-ICU in Japan [10]. However, this study had several limitations, including randomly sampled data from the preimplementation period of the tele-ICU and an unadjusted comparison of clinical outcomes before and after the implementation of the tele-ICU. To date, few studies have examined how hospitalization costs changed after the implementation of the tele-ICU. Furthermore, few studies have evaluated which patient groups would benefit from the tele-ICU.

The present study has two main objectives. First, it aims to investigate the effectiveness of implementing the tele-ICU in Japan. Second, it analyzes the cost of this implementation. Using continuously collected administrative data over an 8-year period, we conducted an interrupted time-series analysis with severity adjustment to evaluate heterogeneity across patient subgroups after tele-ICU implementation.

## 2. Materials and Methods

### 2.1. Study Design, Setting, and Data Source

This retrospective cohort study used routinely collected inpatient administrative data from two Japanese academic hospitals: Showa University Hatanodai Hospital and Showa University Koto Toyosu Hospital. The Human Research Ethics Committee of Showa University approved this study. No information allowing the identification of individual patients, hospitals, or physicians was obtained, and the requirement for informed consent was waived because of the anonymous nature of the data.

This study was performed with Japanese inpatient administrative data, namely, Diagnosis Procedure Combination data, which include discharge abstracts and administrative claims data [11]. The data included the following patient-level data for all hospitalizations: age, sex, diagnoses recorded with International Classification of Diseases Tenth Revision (ICD-10) codes, daily procedures recorded via Japanese medical procedure codes, daily drug administrations, and admission and discharge abstracts.

### 2.2. Tele-ICU Implementation

Showa University Hatanodai Hospital is a tertiary medical education facility in Tokyo, and the tele-ICU supports 38 beds in general ICUs and 7 beds in emergency room (ER) intermediate care (IC). The Hatanodai-general-ICU is a medical, surgical, and cardiac unit with at least a 1:2 nurse–patient ratio and a high-intensity staffing model for intensivists. Hatanodai-ER-IC is an emergency unit with at least a 1:4 nurse–patient ratio and a low-intensity staffing model for intensivists. Showa University Koto Toyosu Hospital is a secondary medical education facility located 15 km away from Showa University Hatanodai Hospital, and the tele-ICU supports 15 beds in medical/surgical ICUs. The Toyosu medical/surgical ICU is a medical and surgical unit with at least a 1:2 nurse–patient ratio and a low-intensity staffing model for intensivists.

The tele-ICU system (eCare Manager 4.1.1; Philips, Amsterdam, The Netherlands) was implemented in all the above ICUs/ICs in April 2018 after the telemedicine staff received initial education and training in the system [10]. The support center, Showa eConnect, was set up at Showa University Hatanodai Hospital. The tele-ICU system supports clinical decision-making through centralized patient information management and real-time physiological severity assessment. The tele-ICU staff consists of a board-certified intensivist, specially trained nurses, and a clerical assistant to the doctor. One nurse is responsible for up to 50 patients. A support center nurse is stationed 24/7. Daily tele-ICU activities included communication with onsite staff and patients using a secured audio–video system on demand and a proactive survey of high-risk or physiologically worsening patients to prevent unfavorable events. Venous thrombosis prophylaxis; stress ulcer prophylaxis; the appropriateness of medication, such as catecholamines, vasopressors, analgesics and sedatives; the recommendation of early mobilization; early enteral feeding; and sepsis management were included in the tasks. Because the role of the tele-ICU involves severity evaluation and advice, tele-ICU physicians did not directly issue medical orders but instead provided recommendations to onsite physicians and only recorded the content of the consultation. In addition, as tele-ICU physicians have expertise in respiratory care and lung-protective ventilation, they performed scheduled and/or on-demand respiratory rounds. Tele-ICU physicians are given full authority in bed placement and transfer to the university hospital ICU.

### 2.3. Study Population

All adult patients admitted to ICUs/ICs from April 2014 to March 2022 were included. There were no exclusion criteria in this study.

### 2.4. Exposure

The exposure was the tele-ICU implementation in April 2018.

### 2.5. Outcome Measures

The primary outcome was in-hospital mortality. The secondary outcomes were ICU mortality, complications, length of stay, length of ICU stay, and hospitalization costs. Complications were defined as ICD-10 codes I110, I130, I132, and I50 (congestive heart failure); I60–I64 (stroke); N17, N18 (end-stage renal disease); I21 and I22 (myocardial infarction); and J13–J18, J690, and J958 (pneumonia). For the length of stay, the stay before ICU admission was not included. The Diagnosis Procedure Combination data include estimated total costs on the basis of reference prices in the Japanese national fee schedule that determine item-by-item prices for consultation, oral drugs, injections, procedures, surgery and/or anesthesia, tests, radiology, hospital fees, diet, and other inpatient services [12]. The calculation of hospitalization costs in this study is based on the aggregate of all estimated costs incurred to treat a patient at a participating hospital.

### 2.6. Statistical Analysis

Interrupted time series analyses were used to compare the trends in outcomes for two separate periods before and after the tele-ICU implementation in April 2018. Interrupted time series analysis is a quasi-experimental design used to evaluate the effect of a population-level health intervention implemented at a clearly defined point in time [13,14,15]. We hypothesized that exposure would cause immediate level changes as well as gradual slope changes in outcomes over time [13]. As one of its strengths, an interrupted time series analysis is generally unaffected by typical confounding variables that are relatively constant or slowly changing under a long-term trend, such as population age distribution or socioeconomic status [13]. However, exposure to tele-ICU implementation could change the ICU population rapidly through actions such as transferring critically ill patients treated in the general ward in the pre-issue period to the ICU in the post-issue period. Therefore, we performed interrupted time series analyses with adjusted outcomes for patient baseline characteristics.

First, we performed linear regression models on outcomes with adjustment for a categorical term for month, a categorical term for each ICU/IC unit, and patient characteristics, including age, male sex, body mass index at admission, Charlson comorbidity index, Japan Coma Scale score at admission, ICU admission classification (elective surgery, emergency surgery, or nonoperative), primary diagnosis, and procedures on the day of ICU admission. These patient characteristics were chosen because previous studies have shown in the Diagnosis Procedure Combination data that these patient characteristics have a high predictive ability for in-hospital mortality, with an area under the receiver operating characteristic curve (AUROC) of 0.898 [16]. For length of stay, length of ICU stay, and hospitalization costs, the above linear regression model was performed on log-transformed data because the outcomes were right skewed.

Next, we estimated the mean adjusted outcomes with their 95% confidence intervals (CIs) for each month via the margins package in STATA. We then plotted the adjusted monthly outcomes from April 2014 (month 1) through March 2022 (month 96). For length of stay, length of ICU stay, and hospitalization costs, estimated values were exponentiated and back-transformed to the original scale for interpretation.

Finally, changes in the mean adjusted outcomes before and during the tele-ICU implementation were evaluated via segmented linear regression with interrupted time series analysis [17]. The equation for the interrupted time series analysis was as follows:

$$Y_{t}=\beta_{0}+\beta_{1}T+\beta_{2}X_{t}+\beta_{3}TX_{t}$$

where $Y_{t}$ is the mean adjusted outcome for each month, T is the month since the beginning of the study period (coded from 1 to 96), and $X_{t}$ is a dummy variable indicating before or after the tele-ICU implementation (coded 0 or 1). In this model, $\beta_{1\;}$ represents the baseline slope before the tele-ICU implementation, $\beta_{2\;}$ represents the immediate level change after the tele-ICU implementation, and $\beta_{3}$ represents the slope change after the tele-ICU implementation compared with the baseline slope before the tele-ICU implementation.

The same analyses used for hospitalization costs were performed for each of the 10 subcategories: consultation, oral drugs, injection, procedure, surgery and/or anesthesia, test, radiology, hospital fee, diet, and other inpatient services.

Continuous variables are presented as the means and standard deviations (SDs) or medians and interquartile ranges (IQRs) as appropriate. Categorical variables are described as numbers and percentages. All reported *p* values were two-sided, and values of *p* < 0.05 were considered statistically significant. All analyses were performed via STATA/MP 17.0 software (StataCorp LLC, College Station, TX, USA).

### 2.7. Subgroup and Sensitivity Analysis

Three subgroup analyses were performed stratified by (i) unit (Hatanodai-general-ICU, Hatanodai-ER-IC, and Toyosu-medical/surgical-ICU), (ii) admission type (elective surgery, emergency surgery, and nonsurgery), and (iii) severity (low-, medium-, and high-). To create a severity category, a logistic regression model was used to estimate the expected in-hospital mortality for each individual, adjusting for categorical terms for each unit and the abovementioned patient characteristics. The performance of this model was evaluated, and the AUROC was 0.933. With reference to previous studies [18,19], less than 2.5% of the predicted in-hospital mortality was classified as low severity, between 2.5% and 25% as medium severity, and greater than 25% as high severity. As a sensitivity analysis, we repeated the interrupted time-series analyses after excluding the COVID-19 pandemic period by restricting the study period to April 2014 through March 2020.

### 2.8. Ethics Approval

The Human Research Ethics Committee of Showa University approved this study (Approval number: 2023-244-B [1 March 2024]). We confirmed that all methods were performed in accordance with the relevant guidelines. We confirmed that the need for informed consent was waived by The Human Research Ethics Committee of Showa University.

## 3. Results

### 3.1. Patient Characteristics

A total of 26,684 patients were eligible during the 8-year study period. Of these, 10,981 (41%) and 15,703 (59%) were before and after the tele-ICU implementation, respectively. The percentages of eligible patients in the Hatanodai-general-ICU, Hatanodai-ER-IC, and Toyosu-medical/surgical-ICU were 15,514 (58%), 5986 (22%), and 5184 (19%), respectively (Table 1). The overall median age was 70 (IQR 57–79) years, and 60% were male. The percentages of patients who underwent elective surgery, emergency surgery, and nonsurgery were 40%, 13%, and 47%, respectively. The most common primary diagnosis was cardiac disease, followed by cancer. The unadjusted in-hospital mortality rates before and after the tele-ICU implementation were 14.8% and 12.6%, respectively.

### 3.2. Results of Interrupted Time Series Analyses

**Figure 1:**
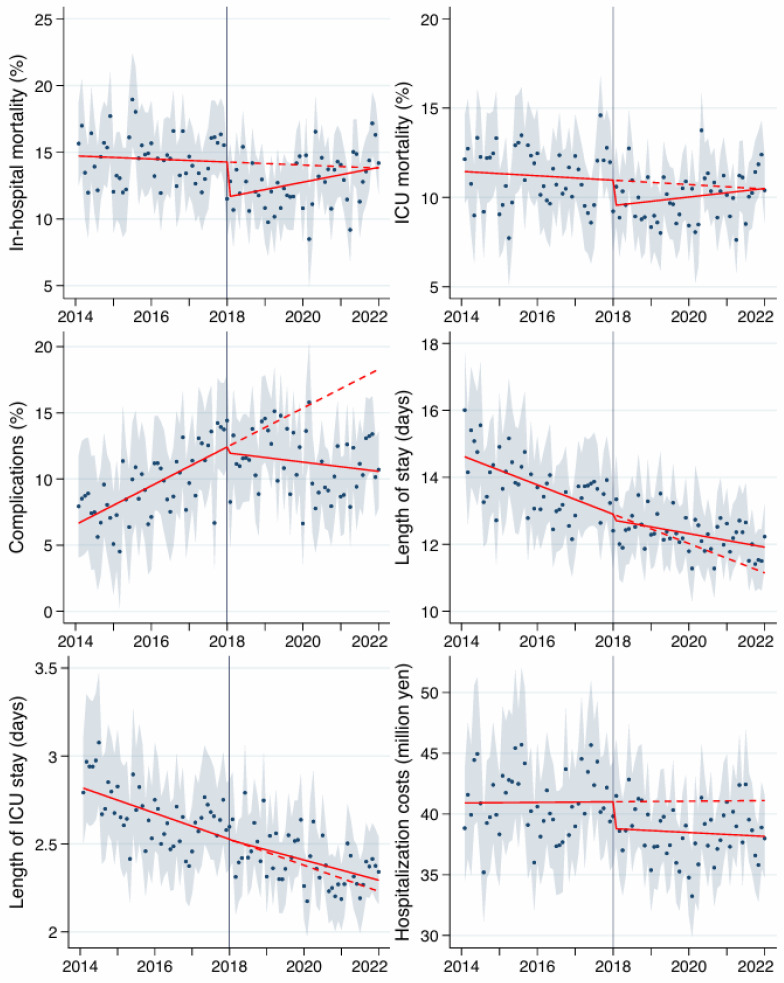
Trends in outcomes before and after tele-ICU implementation. The plots show the mean adjusted outcomes in each month, and the blue areas show their 95% confidence intervals. The outcomes were adjusted with the categorical term for each ICU, age, male sex, body mass index at admission, Charlson comorbidity index, Japan coma scale score at admission, ICU admission classification, primary diagnosis, and procedures on the day of ICU admission. The continuous red lines indicate the baseline trend before the tele-ICU implementation, the level change after the tele-ICU implementation, and the trend after the tele-ICU implementation. The dashed red lines indicate the counterfactual scenarios after the tele-ICU implementation. Each fiscal year started on 1 April and ended on 31 March. ICU, intensive care unit.

There were significant downward level changes for in-hospital mortality (−2.62%; 95% CI −4.08 to −1.16%; *p* value < 0.001) and ICU mortality (−1.42%; 95% CI −2.58 to −0.26%; *p* value = 0.016) after tele-ICU implementation (Table 2 and Figure 1). There was also a significant downward level change for hospitalization costs (−221 thousand yen; 95% CI −412 to −29.7 thousand yen; *p* value = 0.024) after the tele-ICU implementation. There were no significant level changes for complications, length of stay, or length of ICU stay.

There were significant upward slope changes for in-hospital mortality (0.67% per year; 95% CI 0.03–1.30% per year; *p* value = 0.039) and length of stay (0.24 days per year; 95% CI 0.02–0.45; *p* value = 0.029) after tele-ICU implementation. There was a significant downward slope change for complications (−1.82% per year; 95% CI −2.54–−1.09% per year; *p* value < 0.001) after the tele-ICU implementation. There were no significant slope changes for ICU mortality, length of ICU stay, or hospitalization costs.

### 3.3. Subgroup and Sensitivity Analyses

There was a similar downward level change for in-hospital mortality rates as those in the main analyses were observed with statistical significance in the Hatanodai-general-ICU, nonsurgery, and medium-severity groups, with a similar trend observed in the emergency surgery group (Table 3), while the downward level changes in in-hospital mortality were not observed in the Hatanodai-ER-IC, Toyosu medical/surgical-ICU, elective surgery, low-severity, or high-severity groups. The same downward level changes in hospitalization costs as those in the main analyses were observed with statistical significance in the high-severity group, with a trend observed in the Hatanodai-general-ICU, Toyosu-medical/surgical-ICU, emergency surgery, nonsurgery, and low-severity groups but not in the Hatanodai-ER-IC, elective surgery, or high-severity groups. In the sensitivity analysis excluding the COVID-19 pandemic period, the downward level change in in-hospital mortality remained statistically significant.

### 3.4. Medical Costs

The most expensive hospitalization cost category was surgery and/or anesthesia, followed by hospital fees and injection (Table 4). The greatest contributor to the downward level change in overall hospitalization costs was injections (−31.7 thousand yen; 95% CI −54.1 to −9.24 thousand yen; *p* value = 0.006), followed by hospitalization fees (−26.9 thousand yen; 95% CI −51.1 to −2.72 thousand yen; *p* value = 0.029) (Table 5).

## 4. Discussion

We observed significant associations between tele-ICU implementation and lower in-hospital mortality, ICU mortality, and complication rates. Furthermore, we reported a reduction in hospitalization costs of 221 thousand yen per patient immediately after the tele-ICU implementation.

Our results are compatible with previous studies showing a reduction in mortality after tele-ICU implementation [20,21]. With an appropriate methodology, we were able to obtain results similar to those of previous studies, supporting the potential benefit of tele-ICU implementation in Japan. As stated in previous studies [10,22,23], the mechanisms of association with reduced mortality in the tele-ICU may include early intervention in patients whose condition is unstable or deteriorating, continuous and intense monitoring of high-risk patients by skilled staff, quality improvement of care through specialized expertise, refined decision-making processes, and standard medical care due to increased compliance with best practices and guidelines.

In the subgroup analysis, in-hospital mortality decreased significantly in the medium-severity subgroup but not in the low- and high-severity subgroups. Since these patients could subsequently progress into the high-severity group, intensive monitoring, early recognition, and prevention of deterioration by tele-ICU may have been effective in this subgroup. Two observational studies [24,25] reported that the tele-ICU was likely to have little effect on mortality in patients in the low-severity group, similar to our results. The absence of a significant mortality difference in the high-severity group may partly reflect the inclusion of extremely severe cases, such as cardiac arrest on admission in Hatanodai-ER-IC, because outcomes in these critically ill patients may have been difficult to modify. These subgroup findings should be considered exploratory and hypothesis-generating.

In the sensitivity analysis excluding the COVID-19 pandemic period, the association between tele-ICU implementation and lower in-hospital mortality remained statistically significant, although the magnitude of the association was attenuated compared with that in the primary analysis. These findings suggest that the observed reduction in mortality may not be fully explained solely by pandemic-related changes in ICU practice. Nevertheless, residual time-varying confounding factors, including changes in ICU staffing and clinical management over time, may still have contributed to the observed associations.

We observed a significant decreasing trend in complications, which is consistent with the findings of previous studies [24,26]. Possible explanations include continuous monitoring and early intervention, which may improve adherence to best practices and standardized guidelines [26]. However, the sensitivity and specificity of complications extracted from the Diagnosis Procedure Combination (DPC) dataset are low and differ from those reported in previous studies [27]. In particular, the incidence of typical ICU complications, such as ventilator-associated pneumonia or catheter-related bloodstream infection, could not be precisely determined in this study.

### 4.1. Medical Costs

Tele-ICU implementation was associated with an immediate decrease in hospitalization costs of 221 thousand yen (approximately $1480, as 150 yen per $1) per patient. A previous meta-analysis reported that tele-ICU implementation could result in cost savings of $2600–$3000 per patient [28], which is similar to our results. In the subcategory, decreases in injections and hospital fees were the main contributors to the overall cost reduction. One possible explanation is that tele-ICU implementation may have promoted more standardized care management, leading to reduced unnecessary injections and rationalizing ICU or hospital stays. The Japanese acute care system adopted DPC lump-sum payments; therefore, cost reductions in injection and hospital fees could be beneficial, especially for hospital administrators.

However, in the sensitivity analysis excluding the COVID-19 pandemic period, the association between tele-ICU implementation and lower hospitalization costs was no longer statistically significant. These findings suggest that the observed cost reduction may have been influenced in part by pandemic-related changes in ICU utilization, resource allocation, or clinical practice patterns. Therefore, the economic impact of tele-ICU implementation should be interpreted cautiously.

### 4.2. Limitations

There were several limitations in this study. First, this was a retrospective observational study using interrupted time-series analysis and therefore could not establish a robust causal relationship between tele-ICU implementation and the observed outcomes. Although we adjusted for measured patient characteristics and temporal trends, residual confounding may still have remained. Second, the post-implementation period overlapped entirely with the COVID-19 pandemic, which may have affected the ICU case mix, staffing, resource allocation, and clinical practice patterns. Although sensitivity analyses excluding the COVID-19 period were performed, the influence of pandemic-related factors could not be completely excluded. Third, improvements in ICU care over time, including changes in intensivist staffing, respiratory management, sedation practices, and sepsis management, may also have contributed to the observed outcomes. In addition, the role of the tele-ICU team in this study was limited to remote monitoring, severity assessment, and clinical recommendations. Tele-ICU physicians did not directly issue medical orders, and recommendations provided by the tele-ICU team may not always have been implemented by onsite physicians. Therefore, the intensity and actual implementation of tele-ICU interventions could not be fully evaluated. Fourth, this study lacked a contemporaneous control group. Therefore, secular trends in ICU care and other time-varying confounders may not have been fully accounted for despite the interrupted time-series design. Finally, the participating institutions were university hospitals and affiliated hospitals within a single academic network in Japan, which may limit the generalizability of the findings to other healthcare settings.

## 5. Conclusions

This study demonstrated that tele-ICU implementation was associated with lower mortality, fewer complications, and lower hospitalization costs. Further multicenter prospective studies are warranted to assess the robustness of our findings.

## Figures and Tables

**Table 1:** Patient characteristics and outcomes before and after tele-ICU implementation.

Variables	Overall N = 26,684	Before Tele-ICU Implementation N = 10,981	After Tele-ICU Implementation N = 15,703
Unit, n (%)			
Hatanodai-general-ICU	15,514 (58)	7328 (67)	8186 (52)
Hatanodai-ER-IC	5986 (22)	2351 (21)	3635 (23)
Toyosu-medical/surgical-ICU	5184 (19)	1302 (12)	3882 (25)
Age, years, median (IQR)	70 (57–79)	69 (57–78)	71 (56–80)
Male, n (%)	15,935 (60)	6589 (60)	9346 (60)
BMI at admission, kg/m^2^, n (%)			
<18.5	3582 (13)	1398 (13)	2184 (14)
18.5–24.9	14,534 (54)	6111 (56)	8423 (54)
25.0–29.9	4864 (18)	1920 (17)	2944 (19)
≥30.0	1206 (5)	451 (4)	755 (5)
Missing	2498 (9)	1101 (10)	1397 (9)
Charlson comorbidity index, median (IQR)	0 (0–1)	0 (0–1)	1 (0–1)
Japan Coma Scale at admission, n (%)			
Alert	18,637 (70)	7777 (71)	10,860 (69)
Dizziness	3369 (13)	1096 (10)	2273 (14)
Somnolence	1231 (5)	526 (5)	705 (4)
Coma	3447 (13)	1582 (14)	1865 (12)
Admission classification, n (%)			
Elective surgery	10,659 (40)	4694 (43)	5965 (38)
Emergency surgery	3362 (13)	1425 (13)	1937 (12)
Non-surgery	12,663 (47)	4862 (44)	7801 (50)
Primary diagnosis, n (%)			
Cancer	6543 (25)	3095 (28)	3448 (22)
Cardiac diseases	7059 (26)	2864 (26)	4195 (27)
Circulatory diseases other than cardiac	4475 (17)	2016 (18)	2459 (16)
Abdominal disease	1339 (5)	564 (5)	775 (5)
Trauma	1986 (7)	792 (7)	1194 (8)
Others	5282 (20)	1650 (15)	3632 (23)
Procedures at ICU admission, n (%)			
Oxygen supplementation	9232 (35)	2556 (23)	6676 (43)
Mechanical ventilation	4886 (18)	1846 (17)	3040 (19)
Platelet transfusion	1327 (5)	444 (4)	883 (6)
Fresh frozen plasma transfusion	2254 (8)	772 (7)	1482 (9)
Red blood cell transfusion	3280 (12)	1130 (10)	2150 (14)
Dopamine	1241 (5)	794 (7)	447 (3)
Dobutamine	651 (2)	276 (3)	375 (2)
Noradrenaline	4799 (18)	1847 (17)	2952 (19)
Adrenaline	3549 (13)	1357 (12)	2192 (14)
Vasopressin	201 (1)	63 (1)	138 (1)
Cardiac massage	1628 (6)	792 (7)	836 (5)
Mechanical circulatory support	462 (2)	212 (2)	250 (2)
Renal replacement therapy	739 (3)	329 (3)	410 (3)
Outcomes			
In-hospital mortality, n (%)	3606 (13.5)	1626 (14.8)	1980 (12.6)
ICU mortality, n (%)	2810 (10.5)	1314 (12.0)	1496 (9.5)
Complications, n (%)	2827 (11)	915 (8)	1912 (12)
Length of stay, days, median (IQR)	15 (8–28)	15 (9–27)	15 (8–28)
Length of ICU stay, days, median (IQR)	2 (1–4)	2 (1–4)	2 (1–5)
Hospitalization costs, thousand yen, median (IQR)	4050 (1842–10,744)	4015 (1912–10,006)	4088 (1786–11,287)

ICU, intensive care unit; IQR, interquartile range; BMI, body mass index.

**Table 2:** Results of interrupted time series analyses.

Outcomes	Baseline Slope (95% CI)	*p*-Value	Level Change (95% CI)	*p*-Value	Slope Change (95% CI)	*p*-Value
In-hospital mortality, %	−0.11 (−0.56, 0.33)	0.618	−2.62 (−4.08, −1.16)	<0.001	0.67 (0.03, 1.30)	0.039
ICU mortality, %	−0.12 (−0.48, 0.23)	0.499	−1.42 (−2.58, −0.26)	0.016	0.36 (−0.14, 0.87)	0.158
Complications, %	1.47 (0.96, 1.98)	<0.001	−0.44 (−2.11, 1.23)	0.603	−1.82 (−2.54, −1.09)	<0.001
Length of stay, days	−0.44 (−0.59, −0.29)	<0.001	−0.18 (−0.67, 0.31)	0.483	0.24 (0.02, 0.45)	0.029
Length of ICU stay, days	−0.07 (−0.11, −0.04)	<0.001	0.00 (−0.11, 0.10)	0.944	0.02 (−0.03, 0.06)	0.480
Hospitalization costs, thousand yen	2.52 (−56.1, 61.1)	0.933	−221 (−412, −29.7)	0.024	−18.5 (−101, 64.3)	0.662

ICU, intensive care unit and CI, confidence interval. The baseline slope and slope changes were reported in years. The outcomes were adjusted with the categorical term for each ICU, age, male sex, body mass index at admission, Charlson comorbidity index, Japan coma scale score at admission, ICU admission classification, primary diagnosis, and procedures on the day of ICU admission.

**Table 3:** Results of interrupted time series analyses in subgroup and sensitivity analyses.

Outcomes	Baseline Slope (95% CI)	*p*-Value	Level Change (95% CI)	*p*-Value	Slope Change (95% CI)	*p*-Value
Subgroup analyses						
Hatanodai-general-ICU						
In-hospital mortality, %	0.24 (−0.25, 0.72)	0.341	−3.59 (−5.18, −2.01)	<0.001	0.23 (−0.46, 0.92)	0.511
ICU mortality, %	0.06 (−0.29, 0.42)	0.719	−1.32 (−2.48, −0.17)	0.025	−0.09 (−0.59, 0.42)	0.737
Complications, %	1.03 (0.35, 1.72)	0.003	2.99 (0.75, 5.22)	0.009	−1.22 (−2.19, −0.26)	0.013
Length of stay, days	−0.57 (−0.80, −0.34)	<0.001	−0.51 (−1.26, 0.24)	0.186	0.42 (0.09, 0.74)	0.012
Length of ICU stay, days	−0.04 (−0.08, 0.00)	0.080	−0.07 (−0.20, 0.07)	0.323	0.07 (0.01, 0.12)	0.022
Hospitalization costs, thousand yen	66.0 (−41.5, 174)	0.229	−259 (−610, 92.2)	0.148	−36.5 (−189, 115)	0.638
Hatanodai-ER-IC						
In-hospital mortality, %	−0.69 (−1.74, 0.36)	0.198	−0.41 (−3.84, 3.02)	0.814	1.35 (−0.13, 2.84)	0.073
ICU mortality, %	0.00 (−0.88, 0.88)	0.996	−1.77 (−4.64, 1.10)	0.226	0.50 (−0.74, 1.74)	0.428
Complications, %	0.09 (−0.59, 0.77)	0.792	1.73 (−0.49, 3.95)	0.127	−0.79 (−1.75, 0.17)	0.107
Length of stay, days	−0.08 (−0.19, 0.03)	0.136	0.07 (−0.27, 0.42)	0.675	−0.06 (−0.22, 0.09)	0.404
Length of ICU stay, days	−0.09 (−0.15, −0.04)	0.001	−0.23 (−0.41, −0.05)	0.012	0.01 (−0.07, 0.08)	0.887
Hospitalization costs, thousand yen	−7.03 (−33.6, 19.5)	0.603	−54.0 (−141, 32.7)	0.222	0.18 (−37.4, 37.7)	0.993
Toyosu-medical/surgical-ICU						
In-hospital mortality, %	−0.19 (−2.01, 1.64)	0.841	−0.30 (−5.86, 5.25)	0.915	0.79 (−1.14, 2.73)	0.422
ICU mortality, %	−0.50 (−1.45, 0.45)	0.302	1.09 (−1.80, 3.99)	0.458	0.96 (−0.05, 1.97)	0.062
Complications, %	2.44 (−1.66, 6.54)	0.243	−12.8 (−25.3, −0.37)	0.044	−3.06 (−7.41, 1.29)	0.167
Length of stay, days	0.92 (−0.46, 2.31)	0.193	−3.39 (−7.60, 0.83)	0.115	−1.09 (−2.56, 0.38)	0.146
Length of ICU stay, days	0.31 (0.06, 0.56)	0.015	−0.86 (−1.62, −0.10)	0.026	−0.51 (−0.77, −0.24)	<0.001
Hospitalization costs, thousand yen	662 (−51.2, 1375)	0.069	−2087 (−4256, 81.5)	0.059	−736 (−1492, 20.2)	0.056
Elective surgery						
In-hospital mortality, %	−0.08 (−0.31, 0.14)	0.481	0.24 (−0.50, 0.98)	0.521	0.03 (−0.29, 0.35)	0.850
ICU mortality, %	0.05 (−0.06, 0.16)	0.392	0.26 (−0.10, 0.62)	0.163	−0.06 (−0.22, 0.09)	0.434
Complications, %	1.08 (0.34, 1.81)	0.004	−1.80 (−4.20, 0.60)	0.142	−1.2 (−2.23, −0.16)	0.024
Length of stay, days	−0.75 (−0.95, −0.55)	<0.001	0.72 (0.07, 1.38)	0.029	0.37 (0.09, 0.65)	0.010
Length of ICU stay, days	−0.01 (−0.04, 0.03)	0.760	0.02 (−0.10, 0.14)	0.754	−0.01 (−0.06, 0.04)	0.741
Hospitalization costs, thousand yen	82.4 (0.68, 164)	0.048	317 (50.1, 584)	0.020	−2.08 (−118, 113)	0.972
Emergency surgery						
In-hospital mortality, %	−0.03 (−1.15, 1.08)	0.954	−2.49 (−6.12, 1.15)	0.180	0.59 (−0.98, 2.16)	0.463
ICU mortality, %	−0.72 (−1.50, 0.06)	0.071	−0.08 (−2.63, 2.46)	0.949	0.69 (−0.41, 1.79)	0.219
Complications, %	2.38 (0.85, 3.91)	0.002	0.13 (−4.88, 5.13)	0.960	−3.55 (−5.72, −1.38)	0.001
Length of stay, days	−0.86 (−2.26, 0.53)	0.226	−1.53 (−6.10, 3.03)	0.510	1.52 (−0.45, 3.50)	0.131
Length of ICU stay, days	−0.21 (−0.48, 0.06)	0.128	0.07 (−0.80, 0.95)	0.871	0.22 (−0.16, 0.60)	0.254
Hospitalization costs, thousand yen	−228 (−727, 270)	0.369	−1556 (−3184, 72)	0.061	632 (−72.5, 1337)	0.079
Non-surgery						
In-hospital mortality, %	0.08 (−0.69, 0.84)	0.842	−2.89 (−5.39, −0.40)	0.023	0.76 (−0.32, 1.84)	0.166
ICU mortality, %	−0.06 (−0.70, 0.59)	0.866	−1.25 (−3.36, 0.86)	0.247	0.38 (−0.54, 1.29)	0.420
Complications, %	1.42 (0.77, 2.06)	<0.001	−0.33 (−2.44, 1.77)	0.756	−1.75 (−2.67, −0.84)	<0.001
Length of stay, days	−0.12 (−0.29, 0.05)	0.161	−0.01 (−0.56, 0.55)	0.984	−0.04 (−0.28, 0.19)	0.719
Length of ICU stay, days	−0.08 (−0.13, −0.02)	0.005	0.04 (−0.14, 0.23)	0.642	−0.02 (−0.10, 0.05)	0.536
Hospitalization costs, thousand yen	7.84 (−51.7, 67.4)	0.797	−182 (−377, 12.2)	0.066	−80.6 (−165, 3.66)	0.061
Low-severity						
In-hospital mortality, %	−0.15 (−0.36, 0.06)	0.153	0.09 (−0.58, 0.77)	0.791	0.21 (−0.09, 0.50)	0.168
ICU mortality, %	−0.06 (−0.17, 0.05)	0.262	0.12 (−0.25, 0.48)	0.532	0.03 (−0.13, 0.19)	0.723
Complications, %	1.14 (0.51, 1.78)	<0.001	−1.99 (−4.07, 0.08)	0.059	−1.01 (−1.90, −0.11)	0.028
Length of stay, days	−0.64 (−0.84, −0.45)	<0.001	−0.37 (−1.01, 0.26)	0.248	0.35 (0.08, 0.63)	0.012
Length of ICU stay, days	−0.05 (−0.08, −0.02)	0.001	−0.08 (−0.18, 0.02)	0.116	0.03 (−0.02, 0.07)	0.200
Hospitalization costs, thousand yen	−294 (−1072, 485)	0.46	−1605 (−4148, 939)	0.216	840 (−261, 1942)	0.135
Medium-severity						
In-hospital mortality, %	0.43 (−0.28, 1.15)	0.234	−4.44 (−6.77, −2.11)	<0.001	0.55 (−0.46, 1.56)	0.286
ICU mortality, %	−0.11 (−0.65, 0.43)	0.689	−1.33 (−3.10, 0.44)	0.140	0.24 (−0.52, 1.01)	0.533
Complications, %	1.83 (0.80, 2.86)	<0.001	0.20 (−3.17, 3.57)	0.908	−2.56 (−4.02, −1.10)	0.001
Length of stay, days	−0.29 (−0.71, 0.12)	0.164	−0.87 (−2.22, 0.49)	0.209	0.21 (−0.38, 0.79)	0.490
Length of ICU stay, days	−0.14 (−0.24, −0.05)	0.003	−0.03 (−0.34, 0.28)	0.843	0.06 (−0.07, 0.20)	0.362
Hospitalization costs, thousand yen	771 (−754, 2296)	0.322	−6912 (−11,893, −1931)	0.007	−1723 (−3879, 434)	0.117
High-severity						
In-hospital mortality, %	−0.19 (−1.94, 1.55)	0.827	−1.14 (−6.83, 4.55)	0.694	0.39 (−2.07, 2.86)	0.754
ICU mortality, %	0.06 (−1.58, 1.71)	0.94	−1.49 (−6.87, 3.88)	0.586	0.56 (−1.77, 2.89)	0.636
Complications, %	1.59 (0.42, 2.77)	0.008	0.81 (−3.03, 4.65)	0.680	−2.41 (−4.07, −0.74)	0.005
Length of stay, days	−0.12 (−0.27, 0.03)	0.114	0.14 (−0.35, 0.62)	0.578	−0.01 (−0.22, 0.21)	0.962
Length of ICU stay, days	−0.05 (−0.12, 0.02)	0.184	0.15 (−0.08, 0.38)	0.206	−0.05 (−0.15, 0.04)	0.282
Hospitalization costs, thousand yen	−138 (−579, 303)	0.54	−75.9 (−1516, 136 5)	0.918	−263 (−887, 361)	0.408
Sensitivity analysis						
Excluding COVID-19 periods						
In-hospital mortality, %	−0.11 (−0.54, 0.31)	0.596	−1.78 (−3.51, −0.06)	0.043	−0.08 (−1.36, 1.19)	0.897
ICU mortality, %	−0.10 (−0.46, 0.25)	0.57	−0.83 (−2.28, 0.63)	0.265	−0.31 (−1.38, 0.76)	0.567
Complications, %	1.35 (0.84, 1.85)	<0.001	−0.56 (−2.62, 1.49)	0.591	−1.45 (−2.97, 0.07)	0.061
Length of stay, days	−0.39 (−0.55, −0.23)	<0.001	−0.22 (−0.86, 0.42)	0.505	0.26 (−0.21, 0.74)	0.275
Length of ICU stay, days	−0.08 (−0.11, −0.04)	<0.001	−0.02 (−0.16, 0.12)	0.755	0.04 (−0.06, 0.14)	0.458
Hospitalization costs, thousand yen	9.52 (−48.3, 67.4)	0.747	−81.5 (−317, 154)	0.497	−194 (−367, −20.2)	0.029

ICU, intensive care unit and CI, confidence interval. The baseline slope and slope changes were reported in years. The outcomes were adjusted with the categorical term for each ICU, age, male sex, body mass index at admission, Charlson comorbidity index, Japan coma scale score at admission, ICU admission classification, primary diagnosis, and procedures on the day of ICU admission.

**Table 4:** Overall hospitalization costs and their 10 subcategories before and after tele-ICU implementation.

Hospitalization Costs, Thousand Yen, Median (IQR)	Overall N = 26,684	Before Tele-ICU Implementation N = 10,981	After Tele-ICU Implementation N = 15,703
Overall	4050 (1842–10,744)	4015 (1912–10,006)	4088 (1786–11,287)
Subcategory			
Consultation	8 (5–13)	8 (4–12)	9 (6–13)
Oral drugs	43 (9–141)	24 (6–72)	66 (15–196)
Injection	232 (63–982)	228 (66–1082)	235 (60–917)
Procedure	29 (7–98)	25 (8–94)	32 (7–100)
Surgery and/or anesthesia	1752 (0–6007)	1713 (36–5336)	1785 (0–6634)
Test	119 (63–236)	108 (56–211)	128 (68–253)
Radiology	77 (24–176)	82 (21–199)	73 (26–163)
Hospital fees	646 (412–1119)	581 (392–998)	693 (437–1201)
Diet	218 (102–437)	223 (112–427)	214 (93–446)
Others	1 (0–32)	0 (0–31)	6 (0–33)

ICU, intensive care unit and IQR, interquartile range.

**Table 5:** Results of interrupted time series analyses for overall hospitalization costs and their 10 subcategories.

Hospitalization Costs, Thousand Yen	Baseline Slope (95% CI)	*p*-Value	Level Change (95% CI)	*p*-Value	Slope Change (95% CI)	*p*-Value
Overall	2.52 (−56.1, 61.1)	0.933	−221 (−412, −29.7)	0.024	−18.5 (−101, 64.3)	0.662
Subcategory						
Consultation	−0.87 (−1.07, −0.66)	<0.001	−1.01 (−1.69, −0.32)	0.004	2.29 (2.00, 2.59)	<0.001
Oral drugs	3.15 (2.00, 4.3)	<0.001	13.4 (9.66, 17.2)	<0.001	−3.29 (−4.91, −1.66)	<0.001
Injection	−20.7 (−27.6, −13.9)	<0.001	−31.7 (−54.1, −9.24)	0.006	7.66 (−2.05, 17.4)	0.122
Procedure	−3.42 (−4.48, −2.37)	<0.001	1.07 (−2.38, 4.52)	0.543	1.06 (−0.43, 2.56)	0.163
Surgery and/or anesthesia	7.19 (−10.5, 24.9)	0.426	−11.4 (−69.2, 46.4)	0.699	−19.3 (−44.3, 5.74)	0.131
Test	3.08 (0.10, 6.05)	0.043	8.99 (−0.72, 18.7)	0.070	−5.54 (−9.75, −1.33)	0.010
Radiology	−2.43 (−4.07, −0.79)	0.004	−3.67 (−9.04, 1.70)	0.181	5.16 (2.83, 7.48)	<0.001
Hospital fees	10.7 (3.32, 18.1)	0.005	−26.9 (−51.1, −2.72)	0.029	7.03 (−3.44, 17.5)	0.188
Diet	−5.75 (−8.78, −2.72)	<0.001	−6.49 (−16.4, 3.40)	0.198	3.61 (−0.67, 7.89)	0.098
Others	0.29 (0.14, 0.44)	<0.001	−1.07 (−1.56, −0.58)	<0.001	0.07 (−0.14, 0.29)	0.488

CI, confidence interval. The baseline slope and slope changes were reported in years. The outcomes were adjusted with the categorical term for each ICU unit, age, male sex, body mass index at admission, Charlson comorbidity index, Japan coma scale score at admission, ICU admission classification, primary diagnosis, and procedures on the day of ICU admission.

## Data Availability

Although the dataset analyzed in this study is not publicly available, the data that support the findings of this study are available from the corresponding author upon reasonable request.

## Abbreviations

Tele-ICU	telemedicine intensive care unit
ICU	intensive care unit
LOS	length of stay
APACHE IV	Acute Physiology and Chronic Health Evaluation score version IV
ICD-10	International Statistical Classification of Diseases and Related Health Problems version 10
ER	emergency room
IC	intermediate care
AUROC	area under the receiver operating characteristic curve
CI	confidence interval
SD	standard deviation
IQR	interquartile range
DPC	Diagnosis Procedure Combination.
